# Pancreatic and Pancreatic-Like Microbial Proteases Accelerate Gut Maturation in Neonatal Rats

**DOI:** 10.1371/journal.pone.0116947

**Published:** 2015-02-06

**Authors:** Olena Prykhodko, Stefan G. Pierzynowski, Elham Nikpey, Ester Arevalo Sureda, Olexandr Fedkiv, Björn R. Weström

**Affiliations:** 1 Department of Biology, Lund University, Lund, Sweden; 2 Department of Medical Biology, Institute of Rural Health, Lublin, Poland; Charité, Campus Benjamin Franklin, GERMANY

## Abstract

**Objectives:**

Postnatal gut maturation in neonatal mammals, either at natural weaning or after precocious inducement, is coinciding with enhanced enzymes production by exocrine pancreas. Since the involvement of enzymes in gut functional maturation was overlooked, the present study aimed to investigate the role of enzymes in gut functional maturation using neonatal rats.

**Methods:**

Suckling rats (*Rattus norvegicus*) were instagastrically gavaged with porcine pancreatic enzymes (Creon), microbial-derived amylase, protease, lipase and mixture thereof, while controls received α-lactalbumin or water once per day during 14–16 d of age. At 17 d of age the animals were euthanized and visceral organs were dissected, weighed and analyzed for structural and functional properties. For some of the rats, gavage with the macromolecular markers such as bovine serum albumin and bovine IgG was performed 3 hours prior to blood collection to assess the intestinal permeability.

**Results:**

Gavage with the pancreatic or pancreatic-like enzymes resulted in stimulated gut growth, increased gastric acid secretion and switched intestinal disaccharidases, with decreased lactase and increased maltase and sucrase activities. The fetal-type vacuolated enterocytes were replaced by the adult-type in the distal intestine, and macromolecular transfer to the blood was declined. Enzyme exposure also promoted pancreas growth with increased amylase and trypsin production. These effects were confined to the proteases in a dose-dependent manner.

**Conclusion:**

Feeding exogenous enzymes, containing proteases, induced precocious gut maturation in suckling rats. This suggests that luminal exposure to proteases by oral loading or, possibly, via enhanced pancreatic secretion involves in the gut maturation of young mammals.

## Introduction

During postnatal ontogeny, the mammalian gastrointestinal (GI) tract progresses through a transient period of being well adapted for digestion and absorption of maternal milk, the suckling period [1]. Then the gut eventually undergoes a remodelling process to become fully adapted for digestion of the adult diet, the weaning period. Hence, the gut progresses through an inherited genetic program, resulting in growth and vast irreversible structural and functional changes, such as a switch in intestinal brush-border disaccharidases, from lactase to sucrase and maltase, replacement of fetal-type vacuolated enterocytes by adult-type, non-vacuolated enterocytes in the distal small intestine, and decreased enterocyte endocytic activity resulting in “gut closure” as studied in rodents [2,3]. The temporal regulation of these developmental processes is species-dependent [4].

The inherited developmental program can be accelerated precociously by exposure of suckling mammals to the new dietary factors such as spermine or lectin from red kidney beans [5,6]. Despite on the major focus on small intestinal functional maturation the data of such dietary provocation also shows stimulating effects on pancreas function. Our own studies, in neonatal rats, focused on precociously induced gut maturation after exposure to the kidney bean lectin, *phytohaemagglutinin*, showed a stimulating effect on pancreas growth and enzyme content, concomitant with GI tract maturation [6,7]. In addition, studies made in rats showed that injections of the corticosteroids, a well-known hormonal inducer of the intestinal maturation [8] have dramatically increased proteases secretion in the pancreas, similar to which naturally occurs at weaning [9].

The pancreas appears anatomically and morphologically developed at birth, but both the basal and stimulated secretions, especially of enzymes with proteolitic activities, are relatively low during the suckling period in rats and also in pigs [10–12]. The presence of protease inhibitors in colostrum and maternal milk may also decrease the enzyme activity in the gut lumen to protect bioactive milk proteins/peptides from degradation, ensuring their uptake and further transfer into the general blood circulation during suckling period in rats [13,14].

Together these data temped us to investigate if pancreatic enzymes in the gut lumen will contribute to the maturational changes of GI tract in neonatal mammals, having only maternal milk as a diet. To address this, the present study was designed to evaluate the role of the pancreatic enzymes in gut development, using the suckling rat model. This was accomplished by gavage rats with enzymes, using a preparation of porcine pancreatic enzymes Creon which is widely used for enzyme substitution therapy in humans or microbial-derived pancreatic-like enzymes, *i*.*e*., amylase, protease, and lipase. The enzyme treatment was done between 14–16 days of age, *i*.*e*., before normal weaning which naturally occurs between 17–21 days of age, to study effect of enzymes on growth as well as structural and functional development of the GI tract.

## Materials and Methods

### Animals and ethics statement

The experiment was approved by the local Malmö-Lund Ethical Review Committee for Animal Experimentation and conducted in accordance with the European Community regulation concerning the protection of experimental animals (Permit Number: M228–11). To avoid suffering of the animals the exsanguination was done under ketamine anesthesia after checking the eyelid and withdrawal reflex.

This study was carried out using suckling rats (*Rattus norvegicus)* of the Sprague-Dawley strain (Mol: SPRD Han; Taconic M&B, Denmark) that were bred and kept under pathogen-free conditions in the Department Animal facility at Lund University (20 ± 1°C, 50 ± 10 RH%, 12:12 h light-dark cycle). At about 1 week before parturition, the pregnant dams were moved to separate cages (polycarbonate) with aspen wood bedding, enriched with paper-nesting material. The dams were observed daily to establish their parturition date (day 0) and litters with 10–12 pups were used for the study. All rat pups were kept with their dams during the experiments. The rat dams had a free access to water and rodent laboratory chow (RM1, SDS, Essex, England) placed on the lid of cages. In order to prevent the pups from eating the solid chow, the cage height was increased using a 7 cm wall extender.

### Enzymes and enzymes preparations

A pancreatic enzyme preparation, Creon 10000 (Abbott Products GmbH; Hannover, Germany), was used where each capsule is containing 150 mg of pancreatin, extracted from the porcine pancreas and containing mixture of protease, amylase and lipase with activity 4, 53.3 and 66.7 Pharmacopoeia European units (U) per 1 mg of preparation, respectively. The microbial-derived enzymes obtained from Sigma-Aldrich Co, St. Louis, MO, USA: a proteinase with trypsin/chymotrypsin-like activities from *Aspergillius melleus* (type XXIII protease with specific activity ≥ 3000 U/g, where one U hydrolyzes casein equivalent to 1.0 μmole of tyrosine per min at pH 7.5 at 37°C); a lipase, having co-lipase independent activity, from Burkholderia *cepacia* (Amano Lipase PS, with specific activity ≥ 23000 U/g, where one U is defined as the quantity of a standard lipase preparation (Fungi Lipase-International F.I.P. Standard), which liberates the equivalent of 1 μmole of fatty acid from olive oil per minute at pH 7.0 at 37°C) and an alpha-amylase from *Aspergillius oryzae* (with an enzyme activity ≥ 30000 U/g, where one U corresponds to the amount of enzyme which liberates 1 μmol maltose per minute at pH 6.0 and 25°C using starch as substrate). Before each gavage feeding the Creon’s gelatin capsules were opened and enzymes were dissolved in water after removal from coating material in mortar, while all enzymes of microbial origin were dissolved directly in the water prior to stomach gavage.

### Experimental procedure

Three experiments were performed in a split-litter manner where the pups were divided into several weight-matched feeding groups, within each litter. The pups received solutions via a soft stomach tube once a day between 14–16 days of age, with a volume of 0.01 ml per gram body weight (g b.wt) [6].

The first experiment evaluated the effects of pancreatic enzymes of porcine origin. Rat pups from 2 litters were gavaged with either Creon (n = 11), in a dose of 1.5 mg/g b.wt (corresponding to 6 U of protease, 100 U of lipase and 80 U of amylase), or α-lactalbumin (Sigma), 1.5 mg/g b.wt, as control (n = 10) to compensate for the protein content given in the enzyme-treated group.

The second experiment evaluated the effects of different microbial pancreatic-like enzymes and mixtures thereof. Littermates from 3 litters were fed with the individual enzymes in doses recalculated as such, protease 0.5 mg/g b.wt (approx. 8 U), amylase 3.33 mg/g b.wt (approx. 80 U), lipase 0.06 mg/g b.wt (approx. 145 U), as well as a mixture of these enzymes, while water (since no effect of gavaged α-lactalbumin was observed in the 1^st^ experiment) was used in the controls (n = 7 in each group).

In the third experiment the effect of different doses of the microbial protease was studied. Two rat litters were divided into five groups and fed with 2-fold decreasing doses of protease, starting from the effective dose, 0.5 mg/g b.wt (n = 5) and then 0.25 mg/g b.wt (n = 6), 0.125 mg/g b.wt (n = 6), 0.0625 mg/g b.wt (n = 5) (corresponding to approx. 8, 4, 2, and 1 U, respectively), while water (n = 5) was used as the control.

### Absorption test in vivo

In the second experiment, the intestinal macromolecular permeability was tested *in vivo*. The pups at 17 days of age (24 hours after last enzyme feeding to ensure an absence of the exogenous proteolytic enzymes in the lumen) were separated from their dam for 2 hours and then gavage-fed with a macromolecules marker solution, containing bovine serum albumin (BSA, Sigma, 1.25 mg/g b.wt) and bovine IgG, (BIgG, Sigma, 0.25 mg/g b.wt) in a volume of 0.025 ml/g b.wt. Three hours later, 1 ml of blood was collected as described below.

### Sample collection

At day 17, the pups were weighed, sedated with carbon dioxide and anaesthetized by a subcutaneous injection with a mixture of Azaperone (Stresnil, 30 μg/g b.wt; Janssen Pharmaceutica, Beerse, Belgium), and Ketamine (Ketalar, 170 μg/g b.wt; Pfizer, New York, USA) in 0.9% NaCl. The abdomen and thorax were opened and the rats were exsanguinated by blood collection via cardiac puncture, into syringes containing EDTA. After centrifugation at 3000 × g for 15 minutes at 4°C, plasma was collected and immediately frozen at -20°C until further analysis.

The entire pancreas was dissected out, weighed and stored at -70°C. The small intestine (SI) was then removed and after measuring of length was divided into two equal parts, the proximal and distal halves. The intestinal content of each segment was flushed out with ice-cold 0.9% NaCl before the intestinal halves were weighed. The proximal half of the intestine was frozen at -70°C for the enzymes activity analysis, while a 2 cm-long sample from the middle part of distal segment was taken for histology. The stomach was removed, opened and its content was collected for measurement of pH for some of the experiments, and the empty stomach weight was recorded. Then cecum was dissected out, opened, rinsed and weighed.

## Analyses

### Intestinal morphology

All samples for histology were fixed in Bouin’s solution for 24 hours and then stored in 70% ethanol. After dehydration and paraffin embedding, 5 μm thick sections were cut, transferred onto microscope slides, and finally rehydrated and stained with haematoxylin and eosin (H&E), according to standard techniques. The intestinal sections were examined using light microscopy to estimate the ratio of the adult-type (non-vacuolated) enterocytes to the length of the villus using the Image*J* program (NIH, Bethesda, MD, USA).

### Intestinal enzymology

The proximal small intestinal portion was homogenized in ice-cold 0.9% NaCl (1:10 wt/vol) using a glass homogenizer. The disaccharidase activities, *i*.*e*., maltase, sucrase and lactase, were measured by incubating the homogenates with the appropriate disaccharide for 60 min at 37°C, after which the liberated glucose was measured using a glucose oxidase reagent (Sigma-Aldrich), in accordance with the Dahlqvist method [15]. The total protein of the intestinal homogenates was determined by the Lowry method [16], modified for 96-well microplates [10], using purified BSA (Sigma-Aldrich) as the standard.

### Pancreas enzymology

The pancreata were homogenized in ice-cold 0.2 M Tris-HCl buffer + 0.05 M CaCl_2_, pH 7.8 (1:10 wt/vol) using a glass homogenizer, and then centrifuged at 15000 x g for 20 min at 4 C. The amylase activity was analysed in homogenates using ethylidene-*p* nitrophenol-glu7 as the substrate according to the manufacturer’s instructions (Infinity Amylase Liquid Stable Reagent; Thermo Scientific, USA). The lipase activity was measured using the Randox lipase kit with the chromogenic substrate *1*,*2-o-dilauryl-rac-glycero glutaric acid-(6′ methyl resorufin)-ester (Randox Laboratories*, *Northern Ireland)*. After activation with enteropeptidase (Sigma-Aldrich), the trypsin activity in the supernatants was determined spectrophotometrically with a microplate modification [10] of the original method of Fritz *et al* [17], using benzoyl-DL-arginine-4-nitroanilide (BAPNA, Sigma-Aldrich) as the substrate. The activity U was recalculated as amount of enzyme that transforms 1 mol of substrate per minute and presented as U per gram of pancreatic wet weight.

### Plasma concentration of permeability markers

The concentrations of macromolecules marker in the blood plasma were quantified by electroimmunoassay [18]. The measurement of BSA concentration was performed using purified BSA as the standard and rabbit anti-BSA as the precipitating antibody. The BIgG concentration was determined using purified BIgG as the standard and rabbit anti-BIgG as the antibody (all Sigma-Aldrich).

### Stomach pH

The stomach contents were re-suspended with 1 ml of 0.9% NaCl and centrifuged at 3000 x g for 1 minute at 4°C, where after the pH was measured in the supernatants by using a pH-meter.

### Statistical analyses

All data are presented as mean ± standard deviation (SD). Statistical comparisons between each treatment and the control group were performed with one-way ANOVA and Dunnett’s multiple comparison post-test, using Prism v4.0 (GraphPad Software, La Jolla, CA, USA). The results were considered significant when *P* < 0.05.

## Results

### Effects of pancreatic enzymes

**Fig 1:**
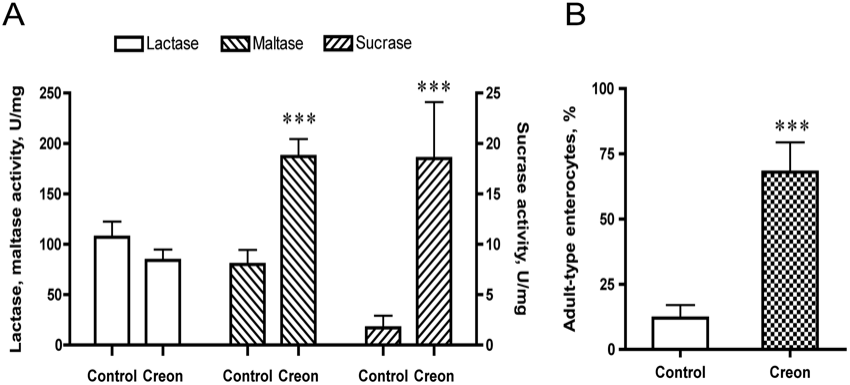
Intestinal disaccharidase activities, *i*.*e*., lactase, maltase and sucrase (U/mg protein), in the proximal small intestine (A), and the portion of adult-type enterocytes, lacking supra-nuclear vacuoles, appearing on the villi in the distal small intestine (B) after gavage feeding the pancreatic enzyme preparation, Creon, or α-lactalbumin (control) during 14–16 days of age in 17 d-old suckling rats. Statistically significant differences between control and enzyme-treated groups (mean ± SD, n = 10–11) are indicated by *** *P* < 0.001.

Oro-gastric gavage with the pancreatic enzyme preparation, Creon, for 3 days had no effect on the body weight gain of the rats, compared to those in the control group given a similar amount of protein, *i*.*e*. α-lactalbumin (Table 1). However, a stimulated growth of the intestines was observed, as manifested by an increased total SI length and weight of both the proximal and distal regions as well as the weight of cecum. An increased pancreas weight was also observed. The intestinal brush-border disaccharidase activities were affected with significantly increased maltase and sucrase activities in the enzyme-treated group (Fig. 1A). Moreover, the villi morphology in the distal small intestine was changed, as seen by the replacement of the fetal-type enterocytes, bearing large supra-nuclear vacuoles, by adult-type enterocytes, lacking such vacuoles (Fig. 1B).

**Table 1:** Body weight and gut organ growth of 17 d-old rats after gavage feeding with pancreatic enzyme preparation, Creon, or α-lactalbumin (control) during 14–16 days of age.

	Body	Stomach	SI	Proximal SI	Distal SI	Cecum	Pancreas	Liver
	weight	weight	length	weight	weight	weight	weight	weight
Treatment	g	mg/g b.wt	cm/g b.wt	mg/g b.wt	mg/g b.wt	mg/g b.wt	mg/g b.wt	mg/g b.wt
Control	40.4 ± 2.5	6.3 ± 0.5	1.5 ± 0.1	17.2 ± 1	15.5 ± 1.3	2.0 ± 0.2	2.7 ± 0.4	33.6 ± 1.5
Creon	39.9 ± 2.0	6.7 ± 0.5	1.7 ± 0.1*	21.7 ± 1.3***	18.7 ± 0.7**	3.2 ± 0.6*	3.2 ± 0.5*	33.8 ± 1.5

The results (mean ± SD, n = 10–11) are presented per gram body weight (g b.wt). Significant differences between enzyme-treated and control groups are indicated by

* *P* < 0.05,

** *P* < 0.01,

*** *P* < 0.001; SI = small intestine.

### Effects of pancreatic-like microbial enzymes

Gavage with the pancreatic-like microbial-derived enzymes resulted in loose stools and a decreased weight gain after the first day for the protease and enzyme mixture groups. However, from the second day of treatment, these groups recovered and showed no differences in body weight at end of the treatment at 17 days (32.2 ± 1.2 g for the protease-treated, 32.5 ± 1.4 g for the enzyme mixture vs. 33.4 ± 1.1 g for the control group). The SI length and weight as well as the cecum weight were significantly increased in pups treated with the enzyme mixture and protease (Table 2). In addition, the stomach pH was lower in these groups as compared to that observed in the control group.

**Table 2:** Body weight, gut organ growth and stomach pH of 17 d-old rats after gavage feeding with microbial-derived enzymes, *i*.*e*., a protease, amylase, lipase, and a mixture thereof, or water (control) during 14–16 days of age.

	Body	Stomach	Stomach	SI	Proximal SI	Distal SI	Cecum	Liver
	weight	weight	pH	length	weight	weight	weight	weight
Treatment	g	mg/g b.wt		cm/g b.wt	mg/g b.wt	mg/g b.wt	mg/g b.wt	mg/g b.wt
Control	33.4 ± 1.1	6.8 ± 0.6	4.3 ± 0.2	1.4 ± 0.1	17.0 ± 2.3	14.5 ± 0.6	2.6 ± 0.5	33.5 ± 3.7
Protease	32.2 ± 1.2	7.2 ± 0.7	3.5 ± 0.3*	1.7 ± 0.1***	24.7 ± 3.5***	17.8 ± 2.5**	4.0 ± 0.5***	35.8 ± 2.5
Amylase	35.5 ± 1.8	6.8 ± 0.4	4.0 ± 0.2	1.5 ± 0.0	16.6 ± 2.0	15.2 ± 2.3	2.5 ± 0.3	33.7 ± 1.3
Lipase	34.3 ± 1.6	7.0 ± 0.8	4.0 ± 0.3	1.5 ± 0.1	17.5 ± 1.7	15.2 ± 1.0	2.4 ± 0.4	34.5 ± 1.8
Mixture	32.5 ± 1.4	7.3 ± 0.5	3.4 ± 0.4*	1.7 ± 0.1***	26.1 ± 2.3***	17.6 ± 0.6***	4.5 ± 0.4***	37.37 ± 2.8*

The results (mean ± SD, n = 7) are presented per grams body weight (g b.wt). Significant differences between enzyme-treated and control groups are indicated by

* *P* < 0.05,

** *P* < 0.01,

*** *P* < 0.001; SI = small intestine.

**Fig 2:**
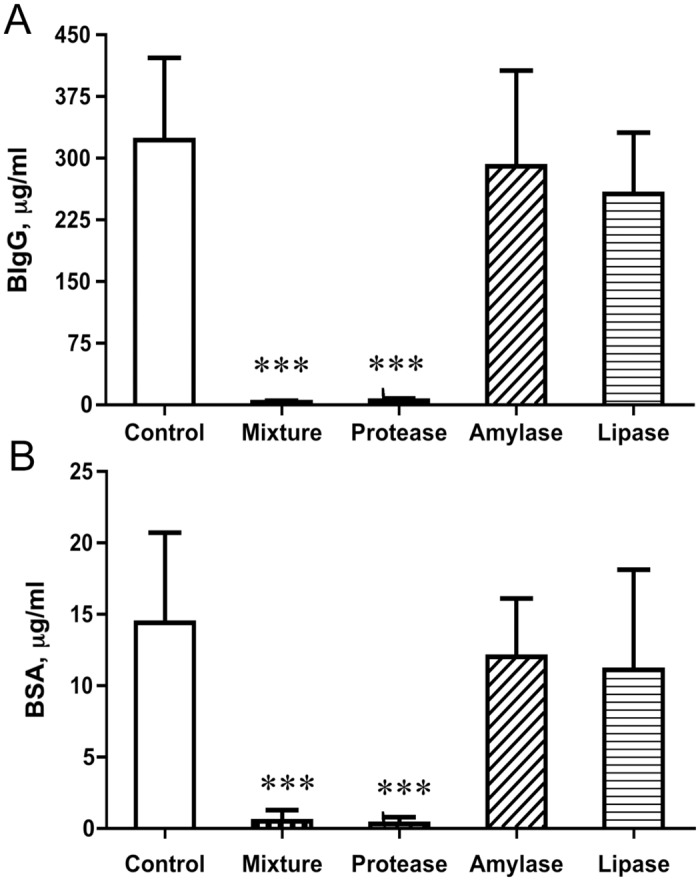
Blood plasma levels of the marker molecules, bovine IgG (BIgG, A) and bovine serum albumin (BSA, B), 3h after their oro-gastric administration to 17 d-old rats that had been treated by gavage feeding with different microbial-derived enzymes, a protease, amylase, lipase, and a mixture thereof, or water (control) during 14–16 days of age. Statistically significant differences between control and enzyme-treated groups (mean ± SD, n = 7) are indicated by *** *P* < 0.001.

**Fig 3:**
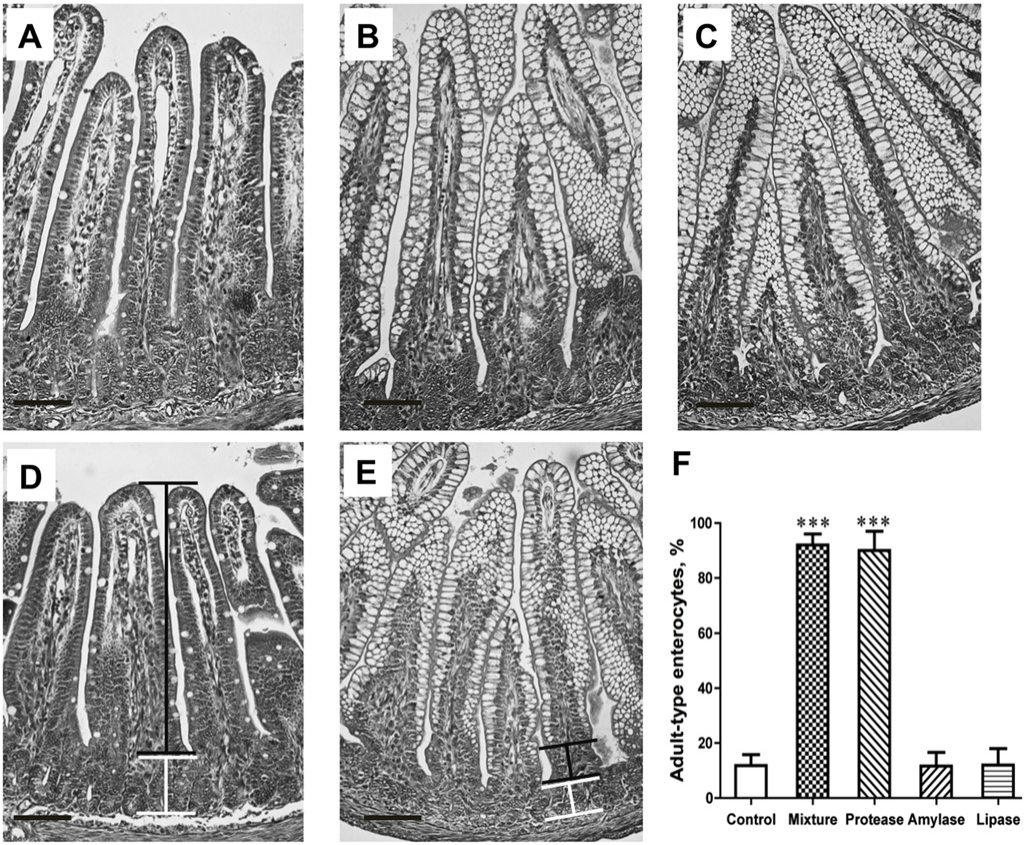
Photomicrographs of H&E stained distal small intestine sections from 17 d-old rats, showing the appearance of mature enterocytes, lacking supranuclear vacuoles, in the villi epithelium after gavage feeding with different microbial-derived enzymes, a protease (A), amylase (B), lipase (C), and a mixture thereof (D) or water (control, E) during 14–16 days of age in suckling rats. The morphometric evaluation of the epithelial maturity, *i*.*e*., the portion of adult-type enterocytes, lacking supranuclear vacuoles, appearing on the villi is shown (F). The horizontal bar inserted indicates 100 μm; black connector line shows the portion of villus with adult-type epithelium, and white line shows the crypt region. Statistically significant differences between control and enzyme-treated groups (mean ± SD, n = 7) are indicated by *** *P* < 0.001.

The absorption of the gavage-fed permeability markers, BSA and BIgG to the blood circulation, was significantly decreased in pups that were treated with the enzyme-mixture and the protease as compared to the control group (Fig. 2). The morphological analysis of the distal part of the SI showed fetal-type enterocytes with large intracellular vacuoles along the villi, from the top to near the base of the villous, in the control group, while almost all these enterocytes were replaced by adult-type, non-vacuolated, enterocytes in the groups treated with the enzymes mixture or the protease (Fig. 3). In addition, the crypt depth was increased for the protease (84 ± 3 μm) and amylase (73 ± 3 μm) groups, compared to controls (62 ± 5 μm, *P* < 0.001). The brush-border disaccharidases activities in the proximal SI were changed in the enzyme mixture and protease-treated groups, showing a decreased lactase activity while increased maltase and sucrase activities, compared to controls (Table 3).

**Table 3:** Intestinal mucosal disaccharidase activities of 17 d-old rats after gavage feeding with microbial-derived enzymes, *i*.*e*., a protease, amylase, lipase, and a mixture thereof, or water (control) during 14–16 days of age.

	Proximal small intestine			
	Protein, mg/g	Disaccharidase activity, U/g		
Treatment		Lactase	Maltase	Sucrase
Control	118.0 ± 22.4	6.1 ± 1.3	6.0 ± 1.8	0.7 ± 0.3
Protease	122.8 ± 23.3	3.4 ± 0.8 ***	15.0 ± 6.7 **	3.4 ± 1.2 ***
Amylase	127.1 ± 27.1	8.0 ± 1.6	7.4 ± 2.3	1.0 ± 0.4
Lipase	123.4 ± 24.4	6.7 ± 1.8	6.9 ± 2.0	1.0 ± 0.5
Mixture	130.6 ± 31.3	4.2 ± 1.2 **	18.5 ± 6.3 ***	3.7 ± 1.1 ***

The results (mean ± SD, n = 7) are presented per gram of wet tissue. Significant differences between enzyme-treated and control groups are indicated by

* *P* < 0.05,

** *P* < 0.01,

*** *P* < 0.001.

The pancreas weight was significantly increased in the enzyme mixture and protease-treated groups. Also, increased amylase and trypsin contents were observed in pancreas in the same groups in comparison to the control pups, while the lipase content remained unchanged (Table 4).

**Table 4:** Pancreas weight and enzyme content of 17 d-old rats after gavage feeding with microbial-derived enzymes, *i*.*e*., a protease, amylase, lipase, and a mixture thereof, or water (control) during 14–16 days of age.

	Pancreas weight	Enzyme activity, U/g		
Treatment	mg/g b.wt	Trypsin	Amylase	Lipase
Control	3.4 ± 0.3	3.3 ± 0.8	19.1 ± 3.6	87046 ± 50700
Protease	4.3 ± 0.4***	6.5 ± 2.4 **	26.4 ± 1.7 **	60369 ± 39526
Amylase	3.5 ± 0.4	3.6 ± 1.2	21.4 ± 4.4	68814 ± 58203
Lipase	3.7 ± 0.5	3.5 ± 1.0	21.3 ± 3.7	60999 ± 46593
Mixture	4.3 ± 0.4***	8.2 ± 2.4 ***	24.8 ± 2.3 **	73331 ± 58037

The organ weights are presented per gram body weight (g b.wt) and enzyme activities per gram of wet tissue (mean ± SD, n = 7). Significant differences between enzyme-treated and control groups are indicated by

** *P* < 0.01,

*** *P* < 0.001.

### Dose-response effects of the protease

**Fig 4:**
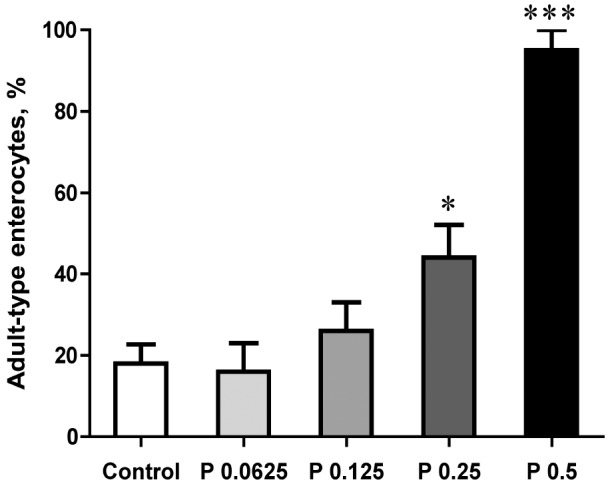
Increased maturity of the small intestinal epithelium of 17 d-old rats, as shown by the portion of adult-type enterocytes, lacking supranuclear vacuoles, appearing on the villi in the distal small intestine after gavage feeding with increasing doses of a microbial-derived protease (P 0.0625, 0.125, 0.25, 0.5 mg per gram body weight) or water (control) during 14–16 days of age. Statistically significant differences between control and enzyme-treated group (mean ± SD, n = 5–6) is indicated by * *P* < 0.05, *** *P* < 0.001.

Oro-gastric feeding with different doses of the microbial-derived protease demonstrated increasing growth of the SI, being significant for the two highest doses (0.5 and 0.25 mg/g b.wt), *i*.*e*., increased length and weight of the SI and weight of the cecum (Table 5). Similarly, the liver and pancreas weights were increased and the stomach pH was decreased with the highest dose (0.5 mg/g b.wt) as compared to the control pups. The morphological analyses of the distal SI for the proportion of adult-type, non-vacuolated enterocytes on the villi, showed a dose-dependent increase, from about 20% in the controls to almost 100% for the highest protease dose given (Fig. 4).

**Table 5:** Gut organ growth and stomach pH of 17 d-old rats after gavage feeding a microbial-derived protease in increasing doses, or water (control) during 14–16 days of age.

	Stomach	Stomach	SI	Proximal SI	Distal SI	Cecum	Pancreas	Liver
	weight	pH	length	weight	weight	weight	weight	weight
Groups	mg/g b.wt		cm/g b.wt	mg/g b.wt	mg/g b.wt	mg/g b.wt	mg/g b.wt	mg/g bwt
Control	6.2 ± 0.2	4.4 ± 0.6	1.4 ± 0.1	15.7 ± 1.6	13.2 ± 1.8	2.3 ± 0.2	3.2 ± 0.2	32.5 ± 2.5
Protease, mg/g b.wt:								
0.0625	6.1 ± 0.3	4.1 ± 0.2	1.5 ± 0.0	16.4 ± 0.8	13.3 ± 1.4	2.3 ± 0.2	3.0 ± 0.4	31.7 ± 1.1
0.125	6.4 ± 0.3	4.1 ± 0.4	1.5 ± 0.1	17.6 ± 1.5	14.7 ± 1.1	2.6 ± 0.31	3.2 ± 0.3	32.8 ± 1.7
0.25	6.3 ± 0.4	3.4 ± 0.7	1.6 ± 0.1**	19.3 ± 2.0**	15.4 ± 1.7*	3.0 ± 0.2***	3.4 ± 0.4	33.0 ± 1.6
0.5	6.9 ± 0.7	3.4 ± 0.6*	1.8 ± 0.1***	28.2 ± 1.7***	18.6 ± 1.0***	4.2 ± 0.1***	5.0 ± 0.2***	38.5 ± 2.4

The organ results (mean ± SD, n = 5–6) are presented per gram body weight (g b.wt). Significant differences between protease-fed and control groups are indicated by

* *P* < 0.05,

**P < 0.01,

*** *P* < 0.001; SI = small intestine.

## Discussion

The present study showed that oro-gastric gavage feeding with preparation of pancreatic enzymes of porcine origin or pancreatic-like enzymes of microbial origin had stimulatory effects on GI growth and accelerated GI maturation in the neonatal rat model. Moreover, it was found that these effects were dependent primarily on the protease component in these preparations.

### Effects on GI growth

The growth promoting effect of the pancreatic and the pancreatic-like enzymes was manifested both as an increased SI length and as an increased weight of the SI and cecum. Previous studies have observed a trophic effect on the SI mucosa due to exposure to pancreatic juice in adult rats [19,20]. The stimulated SI growth in the neonatal rats might be due to an increased crypt cell proliferation, which has been shown to occur during the natural weaning process [21] and during PHA-induced precocious maturation in suckling rats [6,7]. The increased weight of cecum might be due to a direct stimulatory effect of the enzymes but also an indirect one, possibly, due to an altered cecal microflora.

### Effects on GI maturation

In addition to a trophic effect, the enzyme exposure led to maturational changes of the GI tract with a shift in the functional phenotype from the neonatal to the adult type, changes that normally take place during the weaning period at 3 weeks of age, coinciding with the dietary change from milk to solid food [2]. Hence, in the present study the precocious maturation of the stomach was observed with a decreased pH of the stomach content, indicating an enhanced hydrochloric acid secretion before the natural weaning. During natural development this coincides with a switch in enzyme expression from chymosin to increased pepsin production in the stomach mucosa [9,22]. In the present study the switch in SI brush-border disaccharidase activities, *i*.*e*., the change from predominantly lactase to maltase and sucrase activity, manifests the precocious functional maturation of the SI mucosa after enzyme treatment. In addition, we observed an evidently decreased endocytic capability of the SI epithelial cells after enzyme treatments, since in the distal small intestine, the fetal-type enterocytes with their characteristic large supranuclear digestive vacuoles, were replaced by adult-type enterocytes lacking such vacuoles [3]. This change was also coincided with a dramatically decreased intestinal permeability resulted in the ceased macromolecular transfer to the blood circulation, *i*.*e*., “gut closure”. The arrest in absorption of the antibody marker, BIgG, was likely due to the decreased expression of the specific FcRn receptor required for effective transcytosis of IgG antibodies in the proximal SI [23]. The declined transfer of the “non-specific” protein marker, BSA, may also be linked to the FcRn and it’s functional decline, since albumin also has been shown to have a binding capacity to this receptor; however the cessation of macromolecule absorption may also be due to the exchange of cells, having an apical canicular system and supranuclear vacuoles, being involved in protein uptake, with the mature enterocytes lacking these features [3,23,24].

### Protease-induced maturation

In order to identify the effective enzyme(s) in the pancreatic Creon preparation we simplified the system and made a microbial enzyme mixture, containing the major pancreatic-like enzymes, such as amylase, protease and lipase, and tested them together but also separately. The results after gavage with the individual enzymes showed that the trophic as well as the maturation effects were attributed to the protease in a dose-dependent manner, while neither amylase nor lipase had any evident effects on the maturational changes of GI tract. The protease effects appeared to be less prominent when administered alone compared to the effects in the mixture, indicating a greater effect of enzymes when given as a cocktail as they normally release as a mixture in a pancreatic juice. The results indicated that the microbial-derived protease in the high dose could result in some transient gut disturbances, with loose stools and decreased weight gain after the first feeding, while the pancreatic preparation, Creon, did not give such symptoms, may be due to the formulation of enzymes.

### Effects on the exocrine pancreas

Using the neonatal rat model, we have previously demonstrated a stimulating effect of intestinal exposure to PHA on the pancreas, a gut accessory organ, which has no direct contact with the luminal content [6,7]. In the present study we found that enzyme feeding, concomitant with the effects on the GI tract, also had a stimulating effect on the pancreas. The increased pancreas weight may indicate a proliferation of the enzyme producing acinar cells, as was recently reported for the enzyme-treated suckling piglets [25]. Pancreatic functional maturation was verified by the increase in amylase and trypsin content, while the pancreatic lipase remains unchanged. The measurement of the latter enzyme could, however, have been influenced by the method used for measuring of lipase activity, since it is not specific for pancreatic triacylglycerol lipase only, but also for bile-salt stimulated lipase (BSSL), which is highly produced by the pancreas in young rats [26]. Taken together, our results indicate that the immature pancreas appears to respond to luminal exogenous enzymes by changing the functional pattern toward an adult mode, indicating induction of maturation.

### A hypothesized mechanism of enzyme stimulated gut maturation in the neonatal rat

The results obtained in the present study together with an available published data suggest an explanation for the naturally low gut luminal proteolytic activity during the neonatal (immature) period. We hypothesize that the overall low pancreatic function and the high presence of milk enzyme inhibitors is optimal not only to ensure an efficient digestion and absorption of the easy digestible milk nutrients together with a preservation of the bioactive milk components from enzymatic degradation, but also to “maintain” the intestine immature and well adapted to the milk diet during the suckling period.

We show in the present study that feeding neonatal rats with enzymes stimulated GI growth and maturation and speculate that these effects are due to exposure of the SI epithelial cells to proteases. Thus, the observed effects of exposure to exogenous enzyme may mimic the effects of endogenous pancreatic enzymes that increase during the natural weaning process. For instance, Harada *et al* demonstrated that feeding the synthetic trypsin inhibitor, Camostate, to suckling rats, resulted in precocious SI maturation with decreased macromolecular uptake and switched mucosal disaccharidases activities [27,28]. One might think that these findings appear to be opposite to ours, since an protease inhibitor was used to induce maturation, but the data obtained by Kisfalvi *et al*, using also a neonatal rat, showed that orally administered Camostate rapidly stimulates the exocrine pancreas with release of accumulated trypsin to the lumen [29]. Taken together, it is an indication that mainly proteases are involved in the acceleration of SI maturation. Thus, mucosal proteinase-activated receptor (PAR) could, possibly, be the target for the proteases. In fact, PAR-2 receptors are expressed by epithelial cells in the gut mucosa and have been reported to be involved in the regulation of epithelial proliferation and differentiation *in vitro* [30] and will therefore be further studied as possible candidate.

### Conclusion and implications

Our results suggest that pancreatic enzymes are involved in the gut maturation in neonatal rats and the main role appears to belong to the proteases. Apart from pancreatic proteases, exogenous microbial broad-spectrum proteases had similar effects, and may be considered as an alternative for accelerating gut maturation. However, additional studies are necessary to test the effects of the different microbial enzymes, because of possible unfavorable effects due to their formulation or/and impurity. The results obtained in the neonatal rat model are promising and could be further explored for designing a therapy, which might promote the GI maturation in mammalian neonates if needed, *e*.*g*., in pre-term born babies who have impaired gut function due to their immaturity. The concept of improved gut function of mammalian species by the proteases that can be added into the diet, either before or during weaning, could be a possible option to induce GI maturation. For instance, the post-weaning period in husbandry animals, *e*.*g*., in pig production, is often problematic with decreased performance or even loss of young animals due to their immature gut function and inability to deal with solid feed, while the stimulation of the gut maturation would enable the young animals to better cope with the weaning process.
